# Effect of primary balloon angioplasty on draining vein diameter and volume flow in patients with arteriovenous fistula: A cohort study

**DOI:** 10.1016/j.amsu.2022.104426

**Published:** 2022-08-18

**Authors:** R. Suhartono, Suhendro, Harrina Erlianti Rahardjo, Alida Roswita Harahap, Chaidir Arif Mochtar, Akhmadu Muradi, Idrus Alwi, Aida Lydia, Aria Kekalih, Vivian Soetikno, Raflis Rustam

**Affiliations:** a Division of Vascular and Endovascular Surgery, Department of Surgery, Faculty of Medicine, University of Indonesia - Cipto Mangunkusumo Hospital, Jakarta, Indonesia; b Department of Internal Medicine, Faculty of Medicine, University of Indonesia - Cipto Mangunkusumo Hospital, Jakarta, Indonesia; c Division of Urology, Department of Surgery, Faculty of Medicine, University of Indonesia - Cipto Mangunkusumo Hospital, Jakarta, Indonesia; d Department of Clinical Pathology, Faculty of Medicine, University of Indonesia - Cipto Mangunkusumo Hospital, Jakarta, Indonesia; e Division of Cardiology, Department of Internal Medicine, Faculty of Medicine, University of Indonesia - Cipto Mangunkusumo Hospital, Jakarta, Indonesia; f Division of Nephrology and Hypertension, Department of Internal Medicine, Faculty of Medicine, University of Indonesia - Cipto Mangunkusumo Hospital, Jakarta, Indonesia; g Department of Community Medicine, Faculty of Medicine, University of Indonesia, Jakarta, Indonesia; h Department of Pharmacology & Therapeutics, Faculty of Medicine, University of Indonesia, Jakarta, Indonesia; i Division of Vascular and Endovascular Surgery, Department of Surgery, Faculty of Medicine, Andalas University, Padang, Indonesia

**Keywords:** Chronic kidney disease, Arteriovenous fistula, Primary balloon angioplasty, Retrospective cohort study

## Abstract

**Background:**

Chronic kidney disease (CKD) and end-stage kidney disease (ESKD) cause major morbidity and mortality in 10% of the global population with CKD. The most common renal replacement therapy is hemodialysis with arteriovenous fistula (AVF) access. AVF often undergoes maturation failure due to feeding artery and draining vein inadequacy. Mechanical dilatation, such as primary balloon angioplasty (PBA), can overcome AVF maturation failure. The volume flow (VF) and diameter of the draining veins in AVF patients must be known to evaluate the effect of PBA on AVF maturation. This study aims to analyze the impact of PBA on VF and draining vein diameter in ESKD patients undergoing AVF surgery.

**Methods:**

A retrospective cohort clinical trial was conducted at our institution. A total of 75 participants had AVF with an arterial diameter >1.5 mm or vein diameter at the AVF creation site of 2–4 mm. The subjects were divided into 2 groups: the intervention group undergoing PBA (n = 36) and the control group, without PBA (n = 39). PBA was performed using a Mustang ballon (3–6 mm, Medtronic). Follow-ups were conducted at 1 week, 2 weeks, and 6 weeks after AVF creation.

**Results:**

Based on the data, the diameter and VF of the draining veins were significantly larger in the intervention group than in the control group (p < 0.001). Furthermore, we found significant differences in the mean diameter and VF of the draining veins between the control and intervention groups at all stages of examination, from preoperatively to 6 weeks postoperatively (p < 0.001). The strength of the analysis was more than 80%.

**Conclusion:**

PBA can increase the diameter and VF of the draining veins in patients with AVF.

## Highlights

• Mechanical dilatation (i.e., primary balloon angioplasty [PBA]) can prevent arteriovenous fistula (AVF) maturation failure.
• Volume flow and diameter of draining veins in AVF patients must be known to evaluate the effect of PBA on AVF maturation.
• PBA 1.5 × larger than nominal vein size can increase the diameter and volume flow of draining veins in the AVF procedure.
• AVF with PBA 1.5 × larger than nominal vein size had a higher probability of AVF maturation than control.

## 1 Introduction

Chronic kidney disease (CKD) and end-stage kidney disease (ESKD) are the leading causes of morbidity and mortality in both developed and developing countries, and it is estimated that 10% of the global population suffered from CKD in 2015 [1]. The definitive renal replacement therapy for CKD patients is kidney transplantation; however, the most frequently chosen renal replacement modality is hemodialysis [2,3]. According to the Indonesian Renal Registry (IRR), 77,892 ESKD patients actively undergo hemodialysis [4].

The preferred vascular access for hemodialysis is through the creation of an arteriovenous fistula (AVF) [4,5]. Based on the Vascular Access 2006 Workgroup, AVF maturation can be assessed using the “Rule of 6s” criteria [4]. However, AVF has a relatively high rate of maturation failure, reaching only 20–40% in 6 months [5,6]. The causes of AVF nonmaturation are due to inadequate feeding arteries or draining veins. This insufficiency is mainly due to localized stenosis and intimal hyperplasia [[7], [8], [9], [10]]. The dilatation technique is one way of overcoming AVF maturation failure by dilating the veins to improve the channel. The dilatation technique used was balloon angioplasty, of which there are of two types: primary balloon angioplasty (PBA), which is performed before the AVF procedure, and balloon angioplasty maturation (BAM), which is performed serially after the AVF procedure [11,12].

PBA is often performed on small-diameter venous lumens due to the high rate of maturation failure, high reintervention rate, and the need for AVF construction at another location due to failure. In venous diameters larger than 1.9 mm and arterial diameters larger than 1.5 mm, PBA is recommended because it can achieve a maturation success rate >60%; this success rate increases in proportion to the increase in venous diameter [13]. This study aims to determine the effect of PBA on the diameter and volume flow (VF) of the draining vein 1 week after AVF surgery, 2 weeks after AVF surgery, and 6 weeks after AVF surgery, and in ESKD patients undergoing AVF construction surgery.

## 2 Material and methods

The was a retrospective cohort clinical trial conducted at Cipto Mangunkusumo General Hospital in Jakarta, Indonesia, and the network hospitals (Fatmawati Hospital Jakarta, Tangerang District Hospital, and Hermina Depok Hospital, Indonesia). The study was conducted from August 2019 to May 2021. We conducted this research after obtaining ethical clearance from the Health Research Ethics Commission, Faculty of Medicine, University of Indonesia (number: KET-574/UN2.F1/ETIK/PPM.00.02/2021). The study is listed in the Research Registry (number: 7979). Our study is presented in compliance with Strengthening the Reporting of Cohort Studies in Surgery (STROCSS) guidelines [14].

The number of research participants was 75 subjects. The inclusion criteria of this study were:

1. ESKD patients referred to our institution for AVF construction for hemodialysis preparation.
2. Minimal arterial diameter at AVF creation site ≥1.5 mm.
3. Minimal vein diameter at AVF creation site 2–4 mm with absence of stenosis or thrombosis of the draining vein.
4. The patient never had prior AVF surgery on the same arm.

The exclusion criteria included:

1. The presence of sclerotic arteries that exceeded the vessel semicircle.
2. ESKD patients with a diagnosis of established draining vein stenosis or draining vein stenosis identified on duplex ultrasonography or venography.

Research subjects were divided into two groups: the intervention group (who underwent AVF creation with PBA in the draining veins), and the control group (without PBA). Subjects were randomized via block randomization, with each block comprising four members. We took participants from the four hospitals with a proportional number of samples according to the number of patients.

Data were collected from medical records, including patient profile data regarding age, gender, and comorbidities. The patient data included the diameter of the brachial artery, cephalic vein, and VF before surgery in addition to the diameter of the brachial artery, cephalic vein, and venous VF at the time of the creation of the AVF and 1 week, 2 weeks, and 6 weeks after its creation.

### 2.1 Ultrasound examination

Duplex ultrasound examination was performed with the parameters of diameter and VF of the draining veins in the juxtaanastomosis area. The examination was performed immediately after the creation of the AVF, then 1, 2, and 6 weeks after its creation.

We used a LOGIQ e Pro edition ultrasound device (General Electric Healthcare, Shanghai, China) with a high-frequency (4.2–11 MHz) linear probe for the examination. Experienced vascular surgeons who performed the vascular access operations performed scans preoperatively and at 1, 2, and 6 weeks after surgery. Patients sat in a supine position on a pillow, arms extended. The VF of the juxtaanastomotic draining vein, vessel sizes, and morphological differences were documented. The body temperatures of the patients were normal. The temperature in the examination room ranged from 68 °F to 77 °F.

### 2.2 Primary balloon angioplasty

Dilatation was performed shortly before AVF surgery, using a balloon that was dilated to 1.5 times larger than the nominal vein before surgery [15]. Under fluoroscopic guidance, a Mustang Percutaneous Transluminal Angioplasty ballon dilatation catheter (Boston Scientific Way, Marlborough, MA 01752, USA) 3.0–6.0 mm in diameter was introduced and gently inflated to 12 atm of pressure for 60 s from the upper arm to the level of the anastomosis (in brachiocephalic AVF).

### 2.3 Diameter of blood vessels

The diameter was measured as the farthest luminal distance or the distance between two opposite walls of the blood vessel (tunica intima) [16]. The ultrasound technique measured the farthest luminal distance or the distance between two opposite walls of the blood vessel (tunica intima) and was reported in millimeters.

### 2.4 Volume flow

VF was defined as the volume of blood flowing in the AVF per second [16]. It was measured indirectly via a formula:

Cross-sectional area (cm^2^) × Blood flow velocity (mean flow velocity in cm/s).

We obtained cross-sectional area and blood flow velocity from Doppler ultrasound examination.

### 2.5 Data analysis

The data were processed using IBM SPSS software version 21.0 (IBM Corp). The Shapiro–Wilk test was used for the description of numerical data. Normally distributed numerical data are presented as mean with standard deviation, or as median with a minimum and maximum if not normally distributed. Categorical data are presented as amount and percentage. We graphically compared the percentage increase in the draining vein VF data between AVF patients with and without PBA. The comparison test for each interval was an unpaired *t*-test, and the overall trend comparison test was the repeated-measures ANOVA test.

## 3 Results

Of the 88 patients, 13 were excluded from the study because they failed to attend follow-up appointments due to the coronavirus disease 2019 (COVID-19) pandemic (i.e., they were lost to follow-up); therefore, 75 patients were included in the study analysis (Table 1). Of the 75 patients, 36 received AVF with PBA (intervention) and 39 patients received AVF without PBA (control).

**Table 1:** Participant characteristics.

Characteristics			Control (n = 39)	Intervention (n = 36)
Systolic blood pressure (mmHg)			149 (128; 189)	153 (134; 190)
Diastolic blood pressure (mmHg)			79 (65; 104)	78 (68; 96)
Age (years), r(SD)			49 (10)	52 (11)
Gender (male)			10	8
Active smoker (n)			6	3
Arteriovenous fistula			15	15
Arterial diameter before surgery (mm) r(SD)			3.8 (0.9)	4 (0.8)
Arterial flow volume before surgery (ml/min) r(SD)			76.4(79.9)	87.1 (52.3)
Venous diameter before surgery (mm)			2.6 (0.5)	3.2 (0.7)
Venous depth before surgery (mm)			2.6 (1.2; 5.2)	2.4 (1.3; 6.3)
Venous volume flow before surgery (ml/min) r(SD)			10.24 (10.13)	18.61 (21.66)
Balloon diameter (mm)				
	3		0	10
		4	0	14
		5	0	6
		6	0	6

Note: mmHg: millimeter of mercury, r: mean, SD: standard deviation, mm: millimeter.

The mean age of the patients in this study was 49 years in the control group and 52 years in the intervention group. Although there were patients who drop-out, there were no significant differences in baseline characteristics.

Table 2 shows a significant difference in the mean vein diameter over time in the control and intervention groups. There was a significant increase in the mean vein diameter in the control group at 1 week, 2 weeks, and 6 weeks after surgery compared with before surgery (p < 0.001 for all). In the intervention group, there was a significant increase in the mean vein diameter immediately after surgery and at 1 week, 2 weeks, and 6 weeks after surgery compared with before surgery (p < 0.001 for all).

**Table 2:** Analysis of the increase in venous diameter in the control and intervention groups.

Group		n	Venous diameter (mm)	P-value	P-value between groups
Control					
	Before surgery	39	2.65 ± 0.48	**<0.001**^a^	Ref
	Immediately after surgery	39	3.05 ± 0.75		0.081^b^
	1 week after surgery	39	3.96 ± 1.06		**<0.001**^b^
	2 weeks after surgery	39	4.26 ± 1.13		**<0.001**^b^
	6 weeks after surgery	39	5.60 ± 1.09		**<0.001**^b^
**Intervention**					
	Before surgery	36	2.76 ± 0.63	**<0.001**^a^	Ref
	Immediately after surgery	36	3.73 ± 0.76		<0.001^b^
	1 week after surgery	36	4.72 ± 0.86		<0.001^b^
	2 weeks after surgery	36	5.34 ± 0.92		<0.001^b^
	6 weeks after surgery	36	6.61 ± 0.68		<0.001^b^

a Repeated-measures ANOVA test.

b Bonferroni test.

Table 3 shows a significant difference in the mean VF of the draining vein over time in the control group. In addition, there were significant increases in the mean VF of the draining vein immediately after surgery (p < 0.001) and at 1 week (p = 0.001), 2 weeks (p = 0.001), and 6 weeks (p < 0.001) after surgery compared with before surgery. The strength of the analysis with a sample size of 39 subjects in the intervention group was above 80%. There was a significant difference in the mean venous VF over time in the intervention group (Table 3). In addition, there was a significant increase in the mean venous VF immediately after surgery and at 1 week, 2 weeks, and 6 weeks after surgery compared with before surgery (p < 0.001 for all). The strength of the analysis with a sample size of 36 subjects in the intervention group was above 80%.

**Table 3:** Analysis of the increase in venous volume flow in the control and intervention groups.

Group		n	Volume flow	P-value	P-value between groups
Control					
	Before surgery	39	2.65 ± 0.48	**<0.001**^a^	Ref
	Immediately after surgery	39	172 ± 143.8		**<0.001**
	1 week after surgery	39	330 ± 229.0		**0.001**^b^
	2 weeks after surgery	39	442 ± 385.3		**0.001**^b^
	6 weeks after surgery	39	708.7 ± 412		**<0.001**^b^
**Intervention**					
	Before surgery	36	27.05 ± 32.9	<0.001^a^	Ref
	Immediately after surgery	36	425 ± 270.6		**<0.001**^b^
	1 week after surgery	36	771.8 ± 513.6		**<0.001**^b^
	2 weeks after surgery	36	961 ± 552.9		**<0.001**^b^
	6 weeks after surgery	36	1.295 ± 644.1		**<0.001**^b^

a Repeated-measures ANOVA test.

b Bonferroni test.

**Fig. 1:**
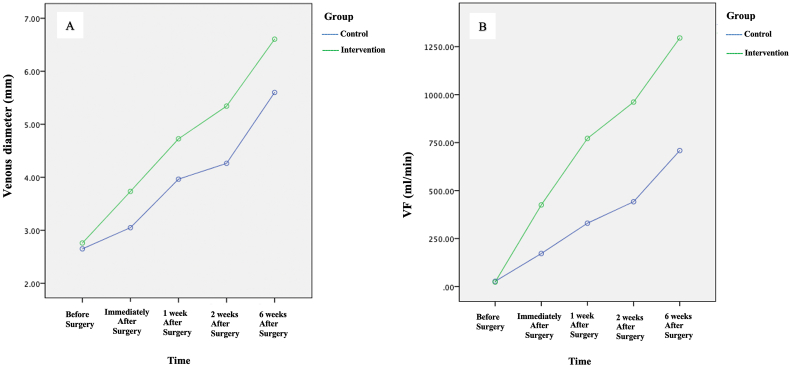
A) Increasing venous diameter between the control and intervention groups over time. B) Increasing the volume of venous flow between the control and intervention groups over time.

Both the control and intervention groups experienced a significant increase in mean vein diameter (p < 0.05) (Table 4). When compared over time between the control and intervention groups, the difference in mean vein diameter changed significantly immediately after surgery (p < 0.001) and at 1 week (p = 0.001), 2 weeks (p < 0.001), and 6 weeks after surgery (p < 0.001). The power of analysis with the large control and intervention groups was more than 80%. Both the control and intervention groups experienced a significant increase in the mean volume of venous flow (p < 0.05). Furthermore, these differences were significant immediately after surgery and at 1 week, 2 weeks, and 6 weeks after surgery (p < 0.001 for all). The analysis power with the large control and intervention sample was more than 80%. The trend of increasing the diameter of the vein and the trend of increasing the volume of venous flow are shown in Fig. 1A and B, respectively.

**Table 4:** Analysis of differences in draining vein diameter and VF between the control and intervention groups.

Group		Control (n = 39)	Intervention (n = 36)	P-value
Venous diameter				
	Before surgery	2.65 ± 0.48	2.76 ± 0.63	0.401
	Immediately after surgery	3.05 ± 0.75	3.73 ± 0.76	**<0.001**^a^
	1 week after surgery	3.96 ± 1.06	4.72 ± 0.86	**0.001**^a^
	2 weeks after surgery	4.26 ± 1.13	5.34 ± 0.92	**<0.001**^a^
	6 weeks after surgery	5.6 ± 1.09	6.61 ± 0.68	**<0.001**^a^
**Volume flow**				
	Before surgery	2.65 ± 0.48	27.05 ± 32.9	0.590
	Immediately after surgery	172 ± 143.8	425 ± 270.6	**<0.001**^a^
	1 week after surgery	330 ± 229.0	771.8 ± 513.6	**<0.001**^a^
	2 weeks after surgery	442 ± 385.3	961 ± 552.9	**<0.001**^a^
	6 weeks after surgery	708.7 ± 412	1.295 ± 644.1	**<0.001**^a^

a Independent *t*-test.

## 4 Discussion

The processed data that were presented in a descriptive graph appeared to indicate a difference in the VF and diameter of the draining veins between the control and intervention groups. However, in the intervention group, the diameter of the draining veins appeared larger than in the control, and the VF of the draining veins was higher than in the control group. The intervention (PBA) group showed an increase in the diameter and VF of the draining veins from the beginning of the procedure to the 6th week. The trend over time seemed to increase and was significant compared to directly.

A study randomized control trial conducted by Veroux et al. [17] reported that the use of balloons in the AVF procedure increased AVF functioning (95%) and resulted in a higher patency (100%) at 6 months compared with no balloon usage. In a prospective study by Khan et al. [18], sixty patients needing surgery for AVF access [but having small cephalic veins (≤2 mm)] were randomly divided into two groups, each with 30 patients. For thirty of these patients, regular hydrostatic dilatation (HD) was performed, whereas, for the other thirty, PBA of the vein was performed before the creation of the fistula. Primary patency, reintervention rates, and maturation timeframes were noted while these patients were monitored for six months. They demonstrated 100% initial efficacy (thrill) and better 6-month patency (93.3%) with PBA than standard HD.

A retrospective study by Garcia et al. [19] and a randomized control trial by Veroux et al. [12] reported that PBA and BAM increased AVF maturation and patency. Venous angioplasty before anastomosis is associated with a low resistance to venous outflow. A prospective observational study by Jemcov [20] on 122 patients assessed the morphology and function of blood vessels in the fabrication of radiocephalic AVF using ultrasound four weeks after surgery; they obtained cut-off diameters of 1.6 mm for the radial artery and 1.8 mm for the cephalic vein as predictors of maturation failure. Masengu et al. [21] conducted a retrospective cohort study on radiocephalic AVF and found that a preoperative radial artery flow volume of 50 ml/min was associated with a sevenfold increase in maturation failure. A retrospective study by Blessions et al. [22] concluded that a radial artery flow volume of 20 ml/min was a significant factor in the failure of radiocephalic AVF maturation despite optimal radial artery diameter. A prospective study conducted by Silva et al. [23] and a systematic review and meta-aggregation of literature from 1966 to January 2015 by Korzadeh et al. [24] recommended the minimum diameter of the radial artery to be 2 mm to provide a high probability of maturation, with a cut-off value of 1.5 mm. There was a significant difference in the preoperative diameter over time and the preoperative flow volume over time; this could be due to the use of balloon dilatation 1.5 times the size of the preoperative diameter. In a prospective study conducted by Zhang et al. [25] using hydrodilatation and the patients were followed for six weeks after AVF, the post-dilation sizes were not measured. Statistical analysis indicated that differences between the control and intervention groups could have been due to unmeasured hydrodilatation, whereas PBA was measurable and more effective.

Our method has enormous potential to increase the number of patients who are eligible for autologous vascular access for hemodialysis by incorporating those who had previously been disqualified for distal autogenous AVF because of small distal cephalic veins. This study has several limitations. The COVID-19 pandemic made patients avoid visiting the hospital and the hospital limited the number of patients until they closed the outpatient department; therefore, some patients were lost to follow-up. Furthermore, we only performed balloon dilatation 1.5 times larger than the nominal vein before surgery; balloon dilatation with another size (e.g., 2 to 2.5 times larger than the nominal vein) would be needed to evaluate the effect of balloon dilatation size on outcomes.

## 5 Conclusions

Using a PBA 1.5 times larger than the nominal vein size increased the diameter and VF of draining veins during the AVF procedure. Based on the diameter and VF of draining vein, AVF with PBA was associated with a higher probability of maturation than without intervention (control). Further studies are needed with more participants and a more extended follow-up time to more precisely observe AVF maturation in ESKD patients.

## Provenance and peer review

Not commissioned, externally peer-reviewed.

## Ethical approval

All procedure for human experiment has been approved by Ethics Commission, Faculty of Medicine, University of Indonesia (number: KET-574/UN2.F1/ETIK/PPM.00.02/2021).

## Sources of funding

No funding or sponsorship.

## Author contribution

R. Suhartono, Suhendro, Harrina Erlianti Rahardjo, Alida Roswita Harahap, Chaidir Arif Mochtar, Akhmadu Muradi, Idrus Alwi, Aida Lydia, and Vivian Soetikno wrote the manuscript and participated in the study design. R. Suhartono, Suhendro, Harrina Erlianti Rahardjo, Alida Roswita Harahap, Chaidir Arif Mochtar, Akhmadu Muradi, Idrus Alwi, Aida Lydia, Vivian Soetikno, and Aria Kekalih drafted the manuscript. R. Suhartono, Suhendro, Harrina Erlianti Rahardjo, Alida Roswita Harahap, Chaidir Arif Mochtar, Akhmadu Muradi, Idrus Alwi, Aida Lydia, Vivian Soetikno, and Aria Kekalih checked the manuscript and made corrections. Aria Kekalih performed bioinformatics analyses and revised the manuscript. Raflis Rustam provided the overall guidance and support. All authors read and approved the final manuscript.

## Trial registry number

This study has been registered with the Research Registry no. 7979

## Guarantor

R. Suhartono and Suhendro.

## Consent

The research was conducted ethically in accordance with the World Medical Association Declaration of Helsinki. The patients have given their written informed consent on admission to use their prospective data base and files for research work.

## Declaration of competing interest

The authors declare that they have no conflict of interests.
